# Effectiveness of treating NELL1-positive idiopathic membranous nephropathy on clinical outcomes, serum cytokine levels (IL-6, IL-10, IL-2, IL-17), nutritional status, and immune responses

**DOI:** 10.5937/jomb0-56892

**Published:** 2025-09-05

**Authors:** Xiaojing Liu, Yanqiu Yu, Kang Li

**Affiliations:** 1 Cangzhou Central Hospital, Department of Nephrology, Cangzhou, Hebei, 061000, China

**Keywords:** glucocorticoids, tacrolimus, NELL, idiopathic membranous nephropathy, nutritional status, inflammatory response, glukokortikoidi, takrolimus, NELL, idiopatska membranozna nefropatija, nutritivni status, inflamatorni odgovor

## Abstract

**Background:**

This study aimed to evaluate the therapeutic effects of glucocorticoids (GCs) combined with tacrolimus (TAC) in the treatment of Neural Epidermal Growth Factor-Like 1 (NELL1)-positive idiopathic membranous nephropathy (IMN), with a focus on clinical outcomes, nutritional status, and inflammatory response.

**Methods:**

A total of 84 NELL1-positive IMN patients from Cangzhou Central Hospital (January 2020-February 2024) were randomly assigned to a research group (GCs+TAC) and a control group (GCs alone). The research group received methylprednisolone (0.5 g), prednisone (0.5 mg/kg), and tacrolimus (0.05 mg/kg/day), while the control group was treated with methylprednisolone and prednisone alone. Clinical parameters, including renal function, blood glucose, lipids, nutritional proteins, and cytokine levels (IL-6, IL-10, IL-2, IL-17), were measured at baseline and after six months of treatment. Nutritional status was assessed using the Nutritional Risk Screening 2002 (NRS 2002).

**Results:**

The two groups had no significant differences in clinical efficacy or adverse reactions. However, the research group exhibited significant improvements in renal function, glycemic control, lipid profile, and nutritional status (P<0.05). Furthermore, the research group showed a more favourable cytokine profile, with more significant reductions in pro-inflammatory cytokines (IL-6, IL-17) and increases in anti-inflammatory cytokines (IL-10, IL-2) compared to the control group (P<0.05). Both groups had comparable safety profiles, with no significant increase in adverse events.

**Conclusions:**

Combining GCs and TAC is an effective and safe therapeutic option for NELL1-positive IMN. It improves clinical outcomes, maintains nutritional stability, and modulates immune responses without increasing adverse reactions.

## Abstract (sr)

**Uvod:**

Ova studija je imala za cilj da proceni terapijske efekte glukokortikoida (GC) u kombinaciji sa takrolimusom (TAC) u lečenju NELL1-pozitivne idiopatske membranozne nefropatije (IMN), s fokusom na kliničke ishode, nutritivni status i inflamatorni odgovor.

**Metode:**

Ukupno su 84 pacijenta sa NELL1-pozitivnom IMN iz Centralne bolnice u Cangžou (januar 2020-februar 2024) nasumično raspoređena u grupu ispitanika (GC+TAC) i kontrolnu grupu (samo GC). Grupa ispitanika je primala metilprednizolon (0,5 g), prednizon (0,5 mg/kg) i takrolimus (0,05 mg/kg/dan), dok je kontrolna grupa lečena samo metilprednizolonom i prednizonom. Klinički parametri, uključujući funkciju bubrega, nivo glukoze u krvi, lipide, nutritivne proteine i nivoe citokina (IL-6, IL-10, IL-2, IL-17), mereni su na početku i nakon šest meseci terapije. Nutritivni status procenjen je pomoću skale Nutritional Risk Screening 2002 (NRS 2002).

**Rezultati:**

Nije bilo značajnih razlika između grupa u pogledu kliničke efikasnosti ili neželjenih reakcija. Međutim, grupa ispitanika pokazala je značajna poboljšanja u funkciji bubrega, kontroli glikemije, lipidnom profilu i nutritivnom statusu (P<0,05). Takođe, grupa ispitanika je imala povoljniji citokinski profil, sa većim smanjenjem proinflamatornih citokina (IL-6, IL-17) i povećanjem antiinflamatornih citokina (IL-10, IL-2) u odnosu na kontrolnu grupu (P<0,05). Obe grupe imale su uporedive bezbednosne profile, bez značajnog porasta neželjenih događaja.

**Zaključak:**

Kombinacija GC i TAC predstavlja efikasnu i bezbednu terapijsku opciju za NELL1-pozitivnu IMN. Poboljšava kliničke ishode, održava nutritivnu stabilnost i moduliše imunske odgovore bez povećanja neželjenih reakcija.

## Introduction

Idiopathic membranous nephropathy (IMN) is one of the most common causes of nephrotic syndrome, especially in middle-aged and elderly individuals. The primary pathological mechanism of IMN involves the diffuse thickening of the glomerular capillary basement membrane due to subepithelial granular deposits of immunoglobulin and complement C3 [1]. Epidemiological data indicate that nephrotic syndrome affects approximately 10.8% of the adult male population worldwide, with IMN contributing to about 20% of these cases [2]. The incidence of IMN is rising, primarily due to the ageing global population [3]. As an autoimmune disorder, IMN involves complex immune responses where maintaining nutritional stability is essential for disease management. Both overnutrition and malnutrition can disrupt renal function and metabolism, potentially contributing to the progression of IMN and other forms of nephropathy [4] [5]. Research by Li Z et al. [4] demonstrated that IMN patients exhibit significant malnutrition compared to the general population, particularly with lower protein levels [6] [7]. Thus, managing IMN requires addressing both the disease pathology and the nutritional status of patients. Neural epidermal growth factor-like 1 protein (NELL1)-positive IMN, a subtype characterised by segmental IgG1 deposition, typically has a higher rate of spontaneous remission [8]. Although NELL1-positive IMN is a type of IMN with a more favourable prognosis, we should also not neglect its treatment.

Of course, we cannot ignore the role of the inflammatory response in NELL1-positive IMN. The family of interleukins (IL) is currently recognised as a major inflammatory factor, and their relationship with IMN has been verified many times [9] [10]. Among them, IL-6 is a pro-inflammatory cytokine typically elevated in autoimmune diseases like IMN [11]. It promotes the differentiation of B cells and the production of acute-phase proteins, contributing to inflammation and glomerular injury. IL-6 is also implicated in developing nephritis, and its elevated levels correlate with disease severity in conditions like lupus nephritis [12]. IL-10, an anti-inflammatory cytokine, is known for its immunosuppressive effects. It inhibits the production of pro-inflammatory cytokines and reduces immune-mediated damage in diseases like IMN. IL-10 has a protective impact on kidney function and is often found to be reduced in autoimmune kidney diseases, suggesting that restoring its levels could improve clinical outcomes [13]. IL-2 is crucial for the activation and proliferation of T cells, especially regulatory T cells (Tregs) [14]. Tacrolimus, an immunosuppressant, inhibits IL-2 production, reducing T-cell activation and less inflammation. This mechanism is key to the effectiveness of tacrolimus (TAC) in treating IMN, as it helps suppress the immune system and prevent further damage to the glomeruli [15]. IL-17 is a potent inflammatory cytokine primarily produced by Th17 cells, and it has been implicated in the pathogenesis of various autoimmune diseases. IL-17 promotes the recruitment of neutrophils and enhances the production of pro-inflammatory cytokines and chemokines. In the context of IMN, IL-17 may contribute to the chronic inflammation and renal damage observed in the disease [16]. This means that these inflammatory cytokines largely determine the progression of IMN.

Glucocorticoids (GCs) remain the mainstay of treatment for IMN, but they are often insufficient to improve clinical symptoms. For a more comprehensive approach, adjunctive immunosuppressive agents (ISs) are commonly used [17]. While ISs enhance therapeutic outcomes, their use can exacerbate nutritional status and result in higher toxicity and side effects when combined with GCs [18]. Combining low-dose GCs with ISs is recommended to reduce these risks, as it ensures the treatment remains effective while minimising adverse effects [19]. However, the clinical effects of low-dose GCs combined with ISs in NELL1-positive IMN remain unclear.

This study evaluates the therapeutic efficacy of low-dose GCs combined with tacrolimus, an IS, in NELL1-positive IMN. In addition, we also focused on the role of interleukins-IL-6, IL-10, IL-2 and IL-17, and these findings will provide new references for future treatment of NELL1-positive IMN.

## Materials and methods

### Sample size calculation

According to the formula for calculating the sample size of a sample survey, n=z^2^×α^2^/d^2^, the confidence interval (z) is set to 90%, the standard deviation (α) is set to 5%, and the margin of sampling error (d) is set to 5%. The sample capacity is calculated to be (n)=81.

### Study population

Based on the sample size calculation, we selected 84 IMN patients treated in Cangzhou City Central Hospital between January 2020 and February 2024 as the study subjects for a randomised controlled trial. Subsequently, using the random number table method, we divided these patients into control and research groups, with 42 cases in each group. Inclusion criteria: Age >18 years old; nephrotic syndrome: 24-hour urine protein (24hUP) >3.5 g and serum albumin (ALB) <30 g/L, with the presence of oedema and/or hyperlipidemia; IMN and NELL1 antigen positive shown by renal biopsy; initial serum creatinine <133 μmol/L; no response to the treatment of serum creatinine level with angiotensin-converting enzyme inhibitor (ACEI)/angiotensin receptor blockers (ARB); no prior therapy with TAC and GCs. Exclusion criteria: Current active infection or autoimmune disease; diabetes, neoplasm, hepatic impairment, or active peptic ulcer disease; secondary IMN, such as lupus nephritis or IMN associated with malignancy; and hepatitis B virus. The Ethics Committee of Cangzhou Central Hospital approved this study (NO. 2022338l), with informed consent obtained from all the subjects. The ethical principles of the Declaration of Helsinki were followed during the research.

### Treatment methods

All treatments were performed in Cangzhou Central Hospital. Control group: Methylprednisolone was given 0.5 g once a day in the first three days of months 1, 3, and 5, followed by oral prednisone at a dose of 0.5 mg/kg every other day. Starting from the 2nd, 4th, and 6th month. Research group: Based on the control group, TAC (0.05 mg/kg/d) was added for treatment, with the plasma concentration maintained at 5–10 ng/mL. The duration of treatment for patients in both groups was 6 months.

### Sample collection and testing

Fasting venous blood was collected at two time points: before treatment (at admission) and after treatment (at the end of the 6-month treatment period). The following tests were performed:

Routine Biochemical Parameters: Total cholesterol (TC), triglycerides (TG), fasting plasma glucose (FPG), blood urea nitrogen (BUN), cystatin C (CysC), low-density lipoprotein cholesterol (LDL-C), high-density lipoprotein cholesterol (HDL-C), haemoglobin (HGB), prealbumin (PA), transferrin (TRF), and total protein (TP) were measured using an automated biochemical analyser (Myers BS-1000M, China).

Cytokine Analysis: Interleukins IL-6, IL-10, IL-2, and IL-17 levels were measured in serum samples using enzyme-linked immunosorbent assay (ELISA), following the manufacturer’s protocols (Wuhan Fine Biotechnology Co., LTD., China). These cytokines were selected due to their critical roles in the immune response and inflammation in IMN.

### Clinical efficacy assessment

The efficacy of the IMN treatment guidelines was evaluated [20]. Complete response: 24hUP <0.3 g or urinary protein/creatinine (uPCR) <300 mg/g, with the presence of renal function symptoms and ALB >35 g/L. Partial response: 0.3 g<24h UP<0.35 g or uPCR within the 3000–3500 mg/g range, or a > 50% reduction in 24hUP after treatment and stable renal function. No response: 24hUP >3.5 g, and a < 50% reduction in 24hUP after treatment compared to before. Recurrence: 24hUP >3.5 g or uPCR >3500 mg/g. Clinical response rate = (complete response+partial response)/total number of patients ×100%.

### Endpoints

(1) Patients’ baseline data (age, gender, course of disease, etc.) were analysed. (2) Efficacy was compared between the two groups. (3) Blood lipids (TC, TG, LDL-C, and HDL-C), blood glucose (FPG), renal function (BUN and CysC), and nutritional proteins (HGB, PA, TRF, and TP) were measured before and after treatment. (4) The malnutrition risk of patients (none, mild, moderate, or severe malnutrition) was assessed using the Nutritional Risk Screening 2002 (NRS 2002) [21] from the dimensions of the severity of the disease, decreased nutritional status, and age. (5) Adverse reactions (infection, diarrhoea, nausea and vomiting, etc.) during the treatment period were statistically counted.

### Statistical analysis

SPSS25.0 (IBM, USA) was used for statistical analysis. All counts were expressed in [n (%)], and the chi-square test was used for comparison between groups; all continuous data were confirmed to conform to a normal distribution using the Shapiro-Wilk test and were recorded as (SIMBOL±s), with inter-group and intra-group comparisons made by independent sample t-tests and paired t-tests, respectively. A value of P<0.05 was considered statistically significant.

## Results

### There was no difference in clinical data

To ensure the reliability of the research results, we first compared patients’ clinical data, including age, sex, body mass index, course of disease, etc., between the two groups. No statistical inter-group significance was identified (P>0.05), confirming comparability (Table 1).

**Table 1:** Comparison of clinical data.

Projects	Control group<br>(n=42)	Research group<br>(n=42)	t (or χ^2^)	P
Age	58.98±6.21	58.88±5.50	0.074	0.941
Male	35 (83.33)	32 (76.19)	0.664	0.415
Female	7 (16.67)	10 (23.81)		
Duration of disease (months)	3.81±0.97	3.60±1.29	0.861	0.392
Body mass index (kg/m^2^)	22.65±1.09	22.41±1.48	0.829	0.41
Systolic blood pressure (mmHg)	139.17±9.94	135.00±11.58	1.769	0.081
Diastolic blood pressure (mmHg)	80.33±5.68	79.48±9.23	0.512	0.61
Family history of disease	2 (4.76)	4 (9.52)	0.718	0.397
No family history of disease	40 (95.24)	38 (90.48)		

### There was no difference in clinical efficacy

Subsequently, the comparison of clinical efficacy showed a clinical response rate of 83.33% in the research group and 76.19% in the control group; the difference in clinical remission rates between the two groups was not statistically significant (P<0.05) (Table 2).

**Table 2:** Comparison of clinical efficacy.

Groups (n=42)	Complete response	Partial response	No response	Clinical response rate
Control	11 (26.19)	21 (50.00)	10 (23.81)	76.19
Research	15 (35.71)	20 (47.62)	7 (16.67)	83.33
χ^2^				0.664
P				0.415

### Symptom improvement was better in the research group

**Figure 1:**
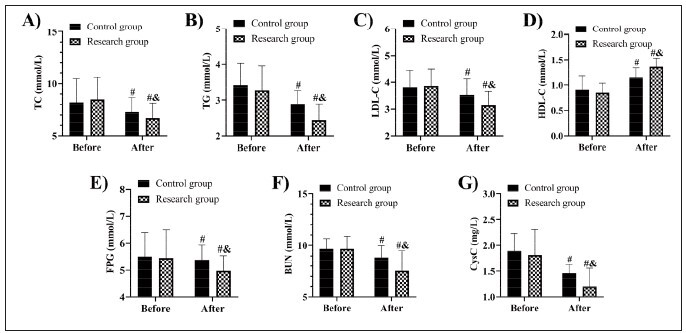
Comparison of A) TC, B) TG, C) LDL-C, D) HDL-C, E) FPG, F) BUN, and G) CysC. vs before treatment ^#^P<0.05, vs control group &P<0.05. Total cholesterol, TC; Triglyceride, TG; Fasting plasma glucose, FPG; Blood urea nitrogen, BUN; Cystatin C, CysC; Low-density lipoprotein cholesterol, LDL-C; High-density lipoprotein cholesterol, HDL-C.

The detection showed no evident inter-group differences in blood lipid, blood glucose, and renal function before treatment (P>0.05). TC, TG, FPG, BUN, CysC, and LDL-C all decreased in both groups after treatment, with more marked decreases in the research group, while HDL-C increased and was higher in the research group compared to the control group after treatment (P<0.05) (Figure 1).

### Nutritional status was better in the research group

**Figure 2:**
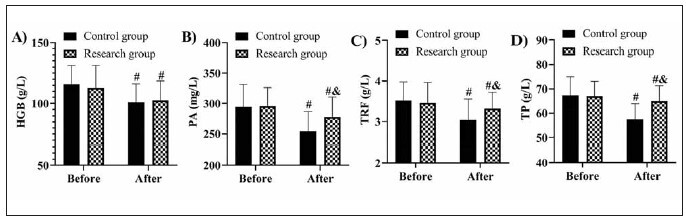
Comparison of A) HGB, B) PA, C) TRF, and D) TP. vs before treatment ^#^P<0.05, vs control group ^&^P<0.05. Haemoglobin, HGB; Prealbumin, PA; Transferrin, TRF; Total protein, TP.

According to the detection results of nutritional proteins, there was no significant inter-group difference before treatment (P>0.05). Both groups showed a decrease in the levels of HGB, PA, TRF, and TP after treatment, with even lower PA, TRF, and TP levels in the research group (P<0.05) (Figure 2).

### The risk of malnutrition was lower in the research group

**Figure 3:**
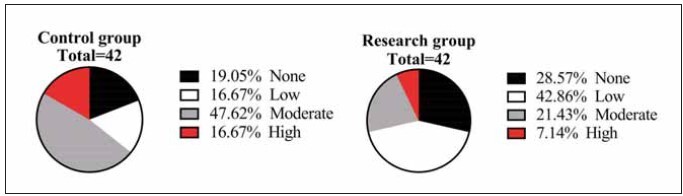
Comparison of NRS2002 findings. Nutritional Risk Screening 2002, NRS 2002.

The results of the NRS2002 survey showed that there was no difference in the comparison of the number of people at no risk of malnutrition and high risk of malnutrition between the two groups (P>0.05) and that there were more people at low risk of malnutrition in the research group than in the control group, while the number of people at intermediate risk of malnutrition was less than in the control group (P<0.05) (Figure 3).

### The inflammatory response was milder in the research group

**Figure 4:**
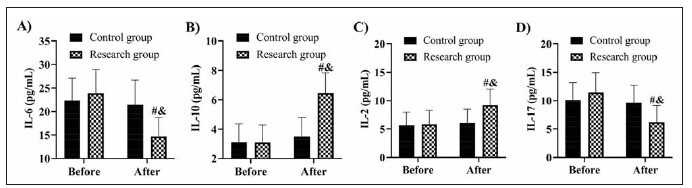
Comparison of A) IL-6, B) IL-10, C) IL-2, and D) IL-17. vs before treatment ^#^P<0.05, vs control group ^&^P<0.05. Interleukin, IL.

The analysis of serum cytokine levels revealed significant changes in the interleukins measured. After 6 months of treatment, the research group showed significantly greater reductions in IL-6 and IL-17 than the control group (P<0.05). Both of these pro-inflammatory cytokines were found to be higher in the research group before treatment, but their levels decreased more substantially in the research group following treatment. On the other hand, IL-2 and IL-10, which are involved in immune modulation and inflammation regulation, showed significant increases in the research group compared to the control group post-treatment (P<0.05). IL-10, known for its anti-inflammatory properties, increased notably in the research group, suggesting an improvement in the regulatory immune response. IL-2, which plays a key role in T-cell activation, also increased, likely reflecting a more robust immune response due to the combination of TAC and glucocorticoid therapy (Figure 4).

### There was no difference in adverse reactions

Finally, the statistical results of adverse reactions showed a total incidence rate of 26.19% in the research group and 19.05% in the control group, with no statistically significant difference (P>0.05) (Table 3).

**Table 3:** Comparison of safety.

Groups<br>(n=42)	Diarrhea	Abnormal blood<br>pressure	Nausea and vomiting	Fever	Dizziness	Total incidence
Control	3 (7.14)	2 (4.76)	2 (4.76)	3 (7.14)	1 (2.38)	26.19
Research	2 (4.76)	1 (2.38)	1 (2.38)	2 (4.76)	2 (4.76)	19.05
χ^2^						0.612
P						0.434

## Discussion

In this study, we found that low-dose GCs combined with TAC helped to improve the symptoms of NELL1-positive IMN patients, improve their nutritional status, and suppress the inflammatory response; these findings provide a new option for the treatment of NELL1-positive IMN in the future.

First, the inter-group comparison of clinical data showed no statistically significant difference, suggesting comparability and high reference value of the results of this study. In terms of clinical efficacy, it can be seen that there is no difference in the clinical remission rate between the two groups. Still, the blood lipids, blood glucose and renal function of the research group are better than that of the control group, indicating that GCs combined with TAC significantly affect NELL1-positive IMN. TAC is a novel calcineurin inhibitor with a narrow therapeutic window and significant individual differences in bioavailability. It is necessary to adjust the dosage of TAC by detecting the blood drug concentration to maximise its therapeutic effect and minimise the incidence of adverse reactions [22]. A study by Zhang X et al. [23] similarly confirmed the significant effect of TAC combined with prednisone in treating primary membranous nephropathy, which is consistent with our view. All the patients in this study used low-dose TAC combined with low-dose prednisone, which can enhance the therapeutic effect while avoiding the adverse reactions caused by high-dose prednisone and TAC. In addition, prednisone can alleviate tubulointerstitial damage caused by TAC, thereby improving patient prognosis and enhancing medication compliance [24]. It is speculated that this is one of the reasons why the renal function of the research group is better than that of the control group after treatment.

Furthermore, due to the suppression of immune function by TAC, long-term use may further worsen the malnutrition of patients [25]. Similarly, as GCs can promote faster metabolism in the body and accelerate the metabolic rate of nutrients, they can lead to endocrine dysfunction, affect the gastrointestinal tract, and easily lead to hyperphagia and gastric nerve hyperactivity, increasing the risk of glucose and lipid metabolism disorders [26]. Malnutrition, as one of the major influencing factors of IMN, affects the progression of IMN and determines the quality of patients’ rehabilitation [27]. In this study, the research group had higher levels of nutritional proteins and a lower risk of malnutrition after treatment, suggesting that GCs combined with TAC are more helpful in maintaining the nutritional status of patients. This is because reducing the dosage of GCs avoids the risk of sustained elevation of blood lipids and immune dysfunction and is more conducive to maintaining the normal metabolism of trace elements in patients [28]. Gawrieh S et al. [29] also confirmed that long-term use of high-dose GCs can lead to the overactivation of their receptors, resulting in the increase of protein synthesis and the acceleration of lipolysis, which is also one of the main reasons for an increased risk of malnutrition in patients. At the same time, GCs themselves have a particular ability to non-specifically inhibit macrophage activity, which can interfere with T lymphocyte-mediated inflammatory responses and, therefore, have a strong immunosuppressive effect [30]. Reducing the dosage can also avoid the risk of further decline in the patient’s immune function and the suppression of immune function caused by TAC. Chen HX et al. [31] also demonstrated that GCs combined with TAC can improve nutritional status in patients with nephrotic syndrome, which is also consistent with our view.

Of course, we also need to focus on the impact of these therapies on the inflammatory response in patients with NELL1-positive IMN. Our analysis of interleukins (IL-6, IL-10, IL-2, and IL-17) revealed significant alterations in cytokine profiles between the two groups. The research group showed a reduction in IL-6 and IL-17, both pro-inflammatory cytokines that play a key role in the pathogenesis of autoimmune diseases and glomerulonephritis. Elevated IL-6 levels have been associated with glomerular injury and progression of renal disease [32], whereas IL-17 is involved in the recruitment and activation of immune cells, contributing to the chronic inflammation seen in IMN [33]. The reduction in these cytokines following treatment may reflect the therapeutic efficacy of GCs and TAC in modulating the immune response in NELL1-positive IMN. IL-10, an anti-inflammatory cytokine, and IL-2, which promotes T-cell activation, showed significantly higher levels in the research group after treatment. These findings suggest that the combination therapy may restore a more balanced immune response, which is essential for limiting inflammation and immune suppression. IL-10’s role in regulating immune responses is well-established, and its increase could indicate a beneficial effect of the treatment in reducing autoimmune activity without compromising the patient’s immune defence [34]. In a study on renal transplantation by Leung ML et al. [35], they also demonstrated that GCs combined with TAC have excellent anti-inflammatory effects, which is consistent with our view. The improved cytokine balance in the research group and the better clinical outcomes and nutritional status highlight the potential of using low-dose GCs combined with TAC to optimise immune modulation in IMN patients. By minimising the adverse metabolic effects of higher doses of these drugs and maintaining immune homeostasis, this combination therapy appears to provide a safer and more effective treatment option for NELL1-positive IMN.

Finally, the inter-group safety comparison showed no significant difference in the incidence of adverse reactions, indicating that GCs combined with TAC do not increase toxicity and side effects and have high clinical safety. The toxic and side effects of GCs on humans have always been a concern by clinical researchers, and how to improve their safety has been the focus of research [36]. In this paper, the adverse reactions of patients with GCs did not increase, which is speculated to be due to the beneficial effects of the treatment regimen on maintaining patients’ nutritional status and the reduced damage to other organs and tissues caused by the reduction of GCs. However, due to the small number of cases in this study, it is not excluded that there may be statistical analysis contingency. Therefore, in the follow-up study, we must also increase the number of cases to verify the study results. At the same time, we also need to add more indicators to evaluate the full effect of low-dose GCs combined with TAC on Nell1-positive IMN.

## Conclusion

Combining GCs and tacrolimus effectively treats NELL1-positive idiopathic membranous nephropathy, improving clinical outcomes, renal function, and nutritional status. This therapy enhances immune modulation and maintains a favourable safety profile. Given its efficacy and minimal adverse effects, this combination therapy offers a promising approach for managing NELL1-positive IMN. However, further studies with larger sample sizes are needed to confirm and refine these findings.

## Dodatak

### Funding

This study was supported by the Medical Science Research Project of Hebei Province (NO.20220338).

### Data availability

Original data in this study are available from the corresponding author upon reasonable request.

### Ethical approval

The study involving human subjects complied with the Declaration of Helsinki and was approved by the ethical committee of the Cangzhou Central Hospital (No. 22021-140-01(z). All participants provided written informed consent.

### Conflict of interest statement

All the authors declare that they have no conflict of interest in this work.
